# Asthma and COPD as co-morbidities in patients hospitalised with Covid-19 disease: a global systematic review and meta-analysis

**DOI:** 10.1186/s12890-023-02761-5

**Published:** 2023-11-22

**Authors:** James Patrick Finnerty, A. B. M. Arad Hussain, Aravind Ponnuswamy, Hafiz Gulzeb Kamil, Ammar Abdelaziz

**Affiliations:** 1 Countess of Chester Hospital NHS Trust, Chester, UK; 2 Department of Respiratory Medicine, Countess of Chester Hospital, Liverpool Road, Chester, CH2 1UL UK; 3 Alexandra Hospital, Worcestershire Acute Hospital NHS Trust, Worcester, UK; 4 University of Chester, Chester, UK

**Keywords:** Asthma, COPD, Chronic obstructive pulmonary disease, COVID-19, SARS-Cov-2

## Abstract

**Background:**

Factors predisposing to increased mortality with COVID-19 infection have been identified as male sex, hypertension, obesity, and increasing age. Early studies looking at airway diseases gave some contradictory results. The purpose of our study was to determine global variation in studies in patients hospitalized with COVID-19 in the prevalence of COPD and asthma; and to determine whether the presence of asthma or COPD affected mortality in the same hospital population.

**Methods:**

A systematic review and meta-analysis of the published literature of COPD and asthma as co-morbidities in patients hospitalized with COVID-19 was performed, looking firstly at the prevalence of these diseases in patients hospitalized with COVID-19, and secondly at the relative risk of death from any cause for patients with asthma or COPD.

**Results:**

Prevalence of both airway diseases varied markedly by region, making meaningful pooled global estimates of prevalence invalid and not of clinical utility. For individual studies, the interquartile range for asthma prevalence was 4.21 to 12.39%, and for COPD, 3.82 to 11.85%. The relative risk of death with COPD for patients hospitalized with COVID-19 was 1.863 (95% CI 1.640–2.115), while the risk with asthma was 0.918 (95% CI 0.767 to 1.098) with no evidence of increased mortality.

**Conclusions:**

For asthma and COPD, prevalence in patients hospitalized with COVID-19 varies markedly by region. We found no evidence that asthma predisposed to increased mortality in COVID-19 disease. For COPD, there was clear evidence of an association with increased mortality.

**Trial registration:**

The trial was registered with PROSPERO: registration number CRD42021289886.

**Supplementary Information:**

The online version contains supplementary material available at 10.1186/s12890-023-02761-5.

## Introduction

Coronavirus 19 (COVID-19) was first reported in December 2019 and has spread to cause a global pandemic. The disease has been described as Coronavirus disease (COVID-19), and the causative virus as Severe Acute Respiratory Syndrome Coronavirus-2 (SARS-Cov-2) [1]. Multiple papers have been published on factors predisposing to serious disease and death from this virus, with particular reference to male sex, hypertension, obesity, and increasing age [2–4].

Three human coronaviruses (HCoVs) can cause pneumonia with fatal outcomes: these are Middle East Respiratory Syndrome Coronavirus (MERS-CoV), which caused an outbreak of respiratory disease in 2012, SARS-CoV, the cause of the SARS outbreak in 2003, and SARS-Cov-2, the cause of the current pandemic: the corresponding diseases are MERS, SARS, and COVID-19. The population affected by MERS had a much higher incidence of preceding chronic lung disease when compared to those affected by SARS-CoV: 13% versus 1.4% [5]. Thus, it is not clear from prior experience of coronavirus outbreaks whether the commonest airway diseases, asthma and COPD, would be expected to predispose to serious disease with SARS-Cov-2. Early in the current epidemic, the opinion was expressed that COVID-19 might cause increased severity of disease in asthmatics [6]. Since that time, multiple studies from most regions of the world have been published.

We addressed the question of whether airways obstruction, either from COPD or asthma, predisposes to worse outcomes in patients hospitalized with COVID-19 disease. We undertook a systematic review of published literature and a meta-analysis. We performed a meta-regression to examine heterogeneity in the prevalence of these diseases in patients hospitalized with proven COVID-19. We determined the following:

1. The regional variation in asthma recorded as a co-morbidity in patients hospitalized with COVID-19 infection.
2. The regional variation in COPD recorded as a co-morbidity in patients hospitalized with COVID-19 infection.
3. The relative risk of asthma in hospitalized patients with COVID-19 who die in hospital compared with those that survive.
4. The relative risk of COPD in hospitalized patients with COVID-19 who die in hospital compared with those that survive.
5. To determine what general conclusions can be made about the risks of asthma and COPD as co-morbidities in patients hospitalized with COVID-19 infection.

## Methods

We performed a systematic review and meta-analysis, with meta-regression on the variables age, sex and region of origin of study. The meta-analysis, meta-regression and calculation of prediction intervals was performed using the software Comprehensive Meta-analysis version 3. The principal analyses were performed used random effects analysis. The studies were weighted using the inverse of the sum of the within study variance and the between studies variance (tau squared, calculated using the method of moments). For clarity of presentation and clinical relevance, the results of the odds ratio analyses were reported after transformation to risk ratios. For the asthma prevalence data, a weighted correlation between prevalence of asthma in hospitalised patients by country in our analysis against published data on national asthma prevalence by country was performed used Stata 17 software. The study was performed in accordance with the Preferred Reporting Items for Systematic Reviews and Meta-analyses (PRISMA) statement [7]. The trial was registered with PROSPERO: registration number CRD42021289886.

### Search strategy and study selection

A search of databases using Healthcare Databases for the NHS in England (HDAS) was performed. The search dates were from the beginning of 2019 until November 2021. For both asthma and COPD, the databases searched were PubMED, Cinahl, and Web of Science, while additional references were sought from previous systematic reviews and meta-analyses.

For asthma: PubMed search using the following search string: ((“covid 19”[MeSH Terms] OR “SARS-CoV2”[Title/Abstract] OR “coronavirus”[MeSH Terms] OR “Novel coronavirus”[Title/Abstract] OR “Coronavirus disease 19”[Title/Abstract] OR “2019-nCoV”[Title/Abstract]) AND (“Hospital Admission”[Title/Abstract] OR “Hospitalisation”[Title/Abstract] OR “Hospitalization”[Title/Abstract]) AND (“asthma”[MeSH Terms] OR “asthma”[All Fields] OR “asthmas”[All Fields] OR “asthma s”[All Fields] OR “Bronchial Asthma”[Title/Abstract] OR “Chronic respiratory disease”[Title/Abstract] OR “Chronic Airway Inflammation”[Title/Abstract])) AND ((humans[Filter]) AND (English[Filter])).

Cinahl: the search term used for CINAHL on the same date was: ((MH “COVID-19”) OR (MH “Coronavirus”) OR (TI “SARS-CoV-2”) OR (AB “SARS-CoV-2”) OR (TI “Novel coronavirus”) OR (AB “Novel coronavirus”) OR (AB “Coronavirus disease 19”) OR (TI “Coronavirus disease 19”) OR (TI “2019-nCoV”) OR (AB “2019-nCoV”)) AND ((TI “Hospital Admission”) OR (AB “Hospital Admission”) OR (TI “Hospitalisation”) OR (AB “Hospitalisation”) OR (TI “Hospitalization”) OR (AB “Hospitalization”) OR (TI “Admitted to hospital”) OR (AB “Admitted to hospital”)) AND ((Asthma) OR (TI “Bronchial Asthma”) OR (AB “Bronchial Asthma”) OR (TI “Chronic respiratory disease”) OR (AB “Chronic respiratory disease”) OR (TI “Chronic Airway Inflammation”) OR (AB “Chronic Airway Inflammation”)).

Web of Science: the following search string to be used in Web of Science: TS = ((COVID-19 OR SARS-CoV-2 OR Coronavirus OR “Novel coronavirus” OR “Coronavirus disease 19”) AND (“Hospital Admission” OR Hospitalisation OR Hospitalization OR “Admitted to hospital”) AND (Asthma OR “Bronchial Asthma” OR “Chronic respiratory disease” OR “Chronic Airway Inflammation”)).

For COPD, the same strategy was used, substituting the following alternatives for asthma: pulmonary disease, chronic obstructive; pulmonary emphysema; bronchitis, chronic (the preceding being MeSH terms used in PubMed); chronic obstructive pulmonary disease; copd; emphysema; and chronic bronchitis.

### Data inclusion and exclusion

The primary eligibility criteria were observational studies including longitudinal studies, cross-sectional studies, prospective and retrospective Cohort studies and case-control studies published in English. Hospitalised adults (> 16 years) regardless of gender or geographical location were eligible for inclusion. SARS-CoV-2 infection had to have been confirmed with reverse transcription - polymerase chain reaction (RT-PCR). People diagnosed with COVID-19 had to have been confirmed on laboratory testing and admitted to hospital. The study population was geographically unrestricted.

Studies which enrolled without RT-PCR confirmation of cases, or included only children’s cases were excluded. Other reasons for study exclusion included: the number of patients with an identified airways disease admitted to the hospital due to COVID-19 was not provided; asthma or COPD patients were grouped together with other chronic respiratory disease patients without extractable data on asthma and COPD; and adults and children part of the same cohort where information on adult population could not be extracted. Other criteria for exclusion were: duplicate publications, clinical trials, case reports, case series, editorials, letters to the editor, reviews, systematic reviews, meta-analyses, unpublished grey literature, and full text not provided in the English language. Studies containing data on groups not able to be generalised to general population (e.g. in pregnant women, chronic renal failure) were also excluded.

### Data extraction

The following data were extracted using a Microsoft Excel template: study name/title, author, year of publication, dates covered by study, country, study design, patient characteristics, mean age, males %, number of participants, number of hospitalised COVID-19 patients, number of hospitalized COVID-19 patients with asthma (or COPD), and number of deaths recorded in those. The information on mean age and sex were specified by protocol to be used as regressors in meta-regression. AP, ABMAH, HK and AA independently performed the searches and AP and JF adjudicated on selection of papers for inclusion in the meta-analysis.

### Quality assessment

The Newcastle-Ottawa quality assessment scale for cohort studies was used: the total score possible for the selection, comparability and outcome domains was eight. AP and JF scored the studies independently, and the final scores were agreed upon by informal discussion.

## Results

**Fig. 1:**
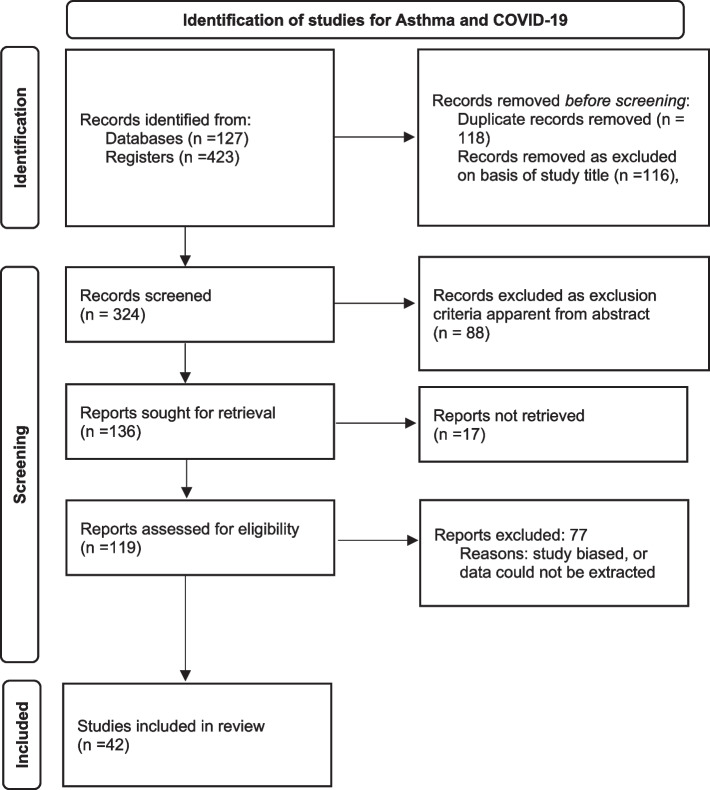
Prisma flow-chart for search on Asthma studies

**Fig. 2:**
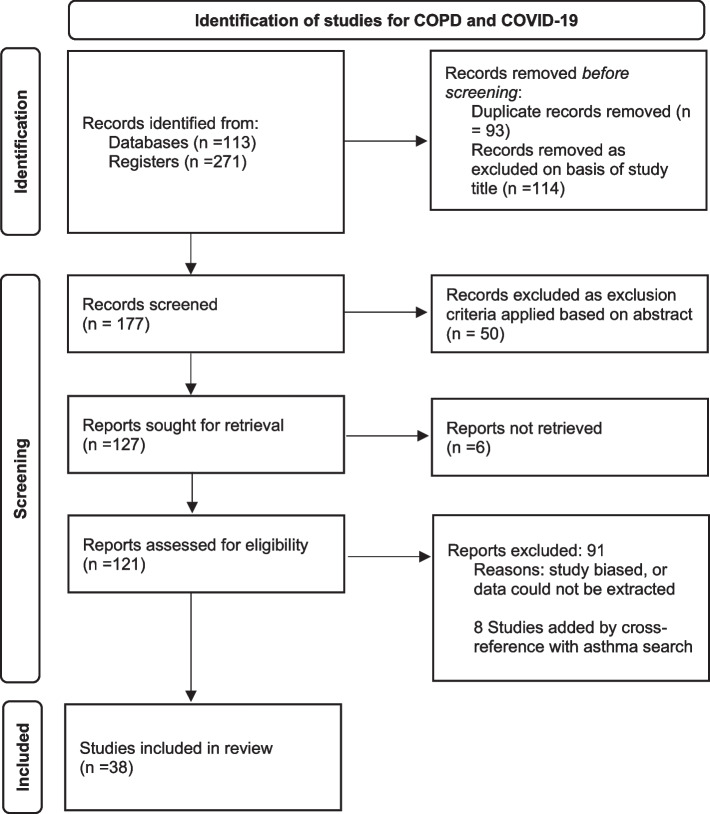
Prisma flow-chart for search on COPD studies

Forty-two studies were identified for inclusion in the analysis of asthma prevalence and mortality, and 38 were identified for inclusion in the analysis of COPD prevalence and mortality. Some studies included data on both asthma and COPD, so that in total 55 studies were included in the analyses [8–62] (Supplementary tables 1 and 2). Using the Newcastle-Ottawa scale, of the 42 studies included for asthma analysis, two studies were given a score of 4, 11 scores of 5, and the remainder were 6 to 8. For the COPD studies, three studies were scored 4, one study was scored 5 and all the rest were 6 to 8. The PRISMA flow charts are provided in Figs. 1 and 2.

### Asthma prevalence

A global prevalence based on a random effects analysis was obtained, of 6.5% (CI 5.5–7.8%). There was clear evidence of heterogeneity, with I^2^ of 99.23%. The point estimate expressed as logit was − 2.659, with tau of 0.580 (tau being the estimate of the SD of the population of true study effect sizes). Assessment for publication bias was performed by inspection of funnel plot and using the technique of Trim and Fill to estimate the effect of any missing studies [63]: asymmetry was evident but this was evident among studies with low standard error and was not a small study effect. The results are shown in Supplementary Fig. 1, which demonstrates marked heterogeneity. Meta-regression using either or both of the variables mean age of study participants and proportion of study participants who were male did not explain any of the variance observed. Meta-regression using sub-region of study origin, with data from all 42 studies, was performed, regressing over the 9 subregions of origin observed in the studies pooled. The test of the model was highly statistically significant: *Q* = 113.27, df = 8, *p* < 0.0001: this indicates that we can reject the null hypothesis of no variance being explained by the covariates; and tau reduced to 0.396. The proportion of between-study variance explained by the model (R^2^ analogue) was 53%. The prevalence of asthma in patients hospitalized with COVID-19 expressed by subregion is given in Table 1, with the difference between the pooled prevalence for North America and East Asia being most marked (*p* < 0.0001).

**Table 1:** Prevalence of asthma in patients hospitalized with COVID-19

Subregion	Number of studies	Random model pooled prevalence (expressed as percentage)	95% Confidence Intervals	*P* for difference from reference subregion
**North America**	**12**	**11.4**	**9.5–13.5**	**Reference region**
**North Europe**	**6**	**12.5**	**10.1–11.3**	**0.59, NS**
**South Europe**	**11**	**4.5**	**3.6–5.5**	< 0.0001*
**Western Europe**	**4**	**9.2**	**5.9–14.0**	**0.33, NS**
**East Asia**	**4**	**1.7**	**0.6–5.1**	< 0.0001*

subregions with 2 or fewer studies excluded. * signifies statistically significant, two tailed at the 5% level

**Fig. 3:**
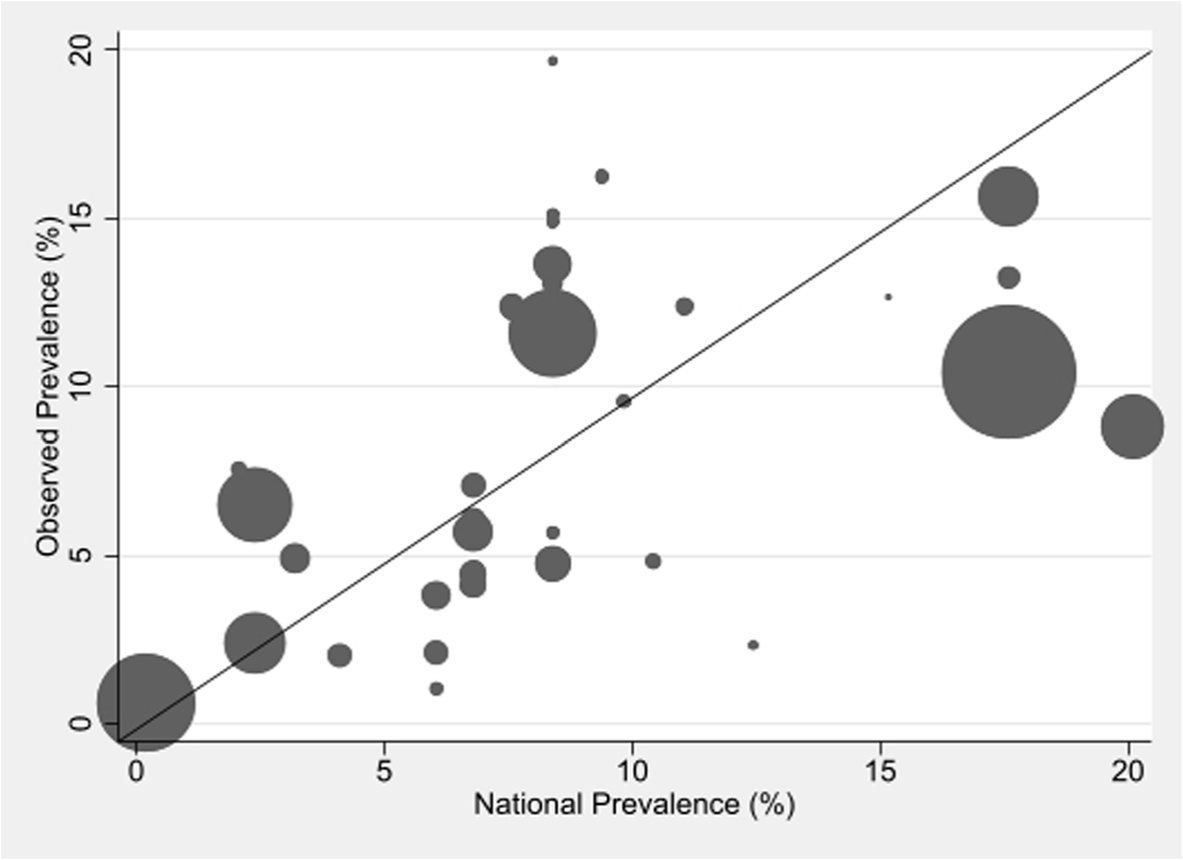
Scatterplot of observed versus expected asthma prevalence. Observed asthma prevalence in 42 individual studies (weighted by number in study) plotted against estimated national adult asthma prevalence. The line is the line of identity

Estimates of expected national asthma prevalence were obtained using the World Health Organisation estimates from their World Health Survey [64] and other sources for the US, Nigeria, Japan, and Korea [65–68]. A regression was performed, assuming no intercept, of the observed values in hospitalized patients in pooled data for each country (using a fixed effects meta-analysis) as the dependent variable, weighing the outcome for each country by the sum of subjects in included studies, against the national prevalence figures as the independent variable, using Stata. Adjusted R^2^ was 0.80 giving R of 0.90 (*p* < 0.0001). The coefficient for the slope of the graph was 0.738 (95% CI 0.736–0.739), suggesting that the prevalence of asthma in hospitalized patients is less than that expected from the population prevalence (Fig. 3).

### Asthma mortality

The analysis on in-hospital all-cause mortality in COVID-19 comparing patients with asthma versus non-asthmatics was conducted on a subset of 17 of the 42 studies above from which the data could be extracted. The prevalence of asthma in studies analysed for mortality (5.8%) was similar to the prevalence in the studies not used in that analysis (7.3%) (*p* = 0.22).

**Fig. 4:**
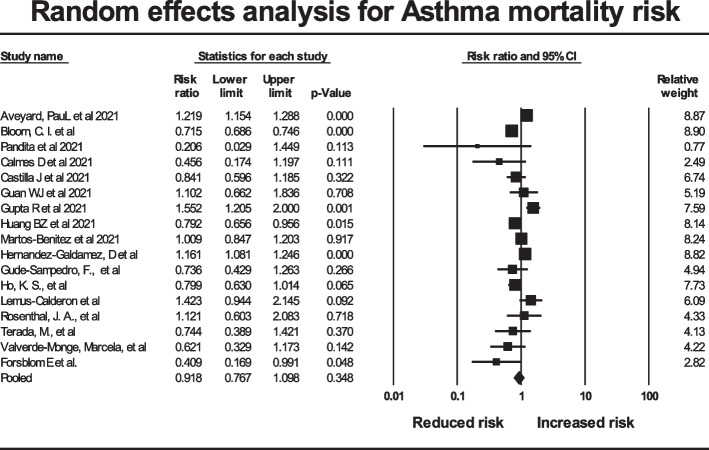
Forest plot for studies in Asthma mortality risk. A risk ratio of 1 indicates no increased mortality risk with asthma. Risk ratios greater than 1 represent increased risk. The black diamond gives the pooled risk ratio and the width of the 95% confidence interval

Meta-regression showed no significant effect of subregions (test of the model: *Q* = 1.76, df = 5, *p* = 0.88), and the same was true for mean age and sex ratio. Analysis of the data by random effects analysis showed a pooled risk ratio of 0.918 (95% CI 0.767 to 1.098), *p* = 0.348, with no evidence of asthma predisposing to increased mortality (Fig. 4). The prediction interval (the true risk ratio for 95% of comparable studies) lies in the interval 0.46 to 1.81.

Assessment for publication bias was performed by inspection of funnel plot as above. The results of shown in supplementary Fig. 2. With studies imputed to the right of the mean, the point estimate for the pooled point estimate for risk ratio remained less than 1 (or log risk ratio less than 0, which is the equivalent). No important publication bias was identified.

Meta-regression of the asthma studies for mortality was performed, using age, proportion male, and UN subregion of study as regressors. Using UN sub-regions alone, the model was not statistically significant: *Q* = 1.76, df 5, *p* = 0.88, *R*^2^ analogue = 0%. Similarly negative results were obtained for the other regressors.

### COPD prevalence

The pooled effect size from 38 studies using a random effects model gave a point estimate for the prevalence of 6.6% (95% CI 5.5 to 7.8%) (*p* < 0.001). This can be expressed as the logit event rate, giving a point estimate of − 2.651, I^2^ of 99.4%, and tau (SD of underlying true distribution of global studies separate from sampling error) of 0.559. There is therefore considerable heterogeneity in the outcomes of globally distributed studies estimating prevalence of COPD in patients hospitalized with COVID-19 (see funnel plot in Supplementary Fig. 3). A meta-regression using sub-region of origin of the studies was performed (Table 2). The model gave statistically significant coefficients (*p* < 0.0001). Tau fell to 0.4232. The proportion of between-study variance explained by the model (R^2^ analogue) was 43%.

**Table 2:** Prevalence of COPD in patients hospitalized with COVID-19

Subregion	Number of studies	Random model pooled prevalence (expressed as percentage)	95% Confidence Intervals	*P* for difference from reference subregion
**North America**	**10**	**10.4**	**7.3–14.5**	**Reference region**
**North Europe**	**6**	**7.0**	**5.6–8.7**	**0.0145** ^*^
**South Europe**	**10**	**7.6**	**5.9–9.6**	**0.0774**
**Western Europe**	**4**	**9.9**	**7.4–13.0**	**0.8463**
**East Asia**	**6**	**1.4**	**0.9–2.1**	0.0001^*^
**Central America**	**2**	**6.5**	**2.6–15.4**	**0.1256**

^*^Signifies statistically significant, two tailed at the 5% level

### COPD mortality

The analysis on in-hospital all-cause mortality in COVID-19 comparing patients with COPD versus non-COPD patients was conducted on a subset of 20 of the 38 studies above from which the data could be extracted. The prevalence of COPD in studies analysed for mortality (7.5%) was similar to the prevalence in the studies not used in that analysis (5.6%) (*p* = 0.11).

**Fig. 5:**
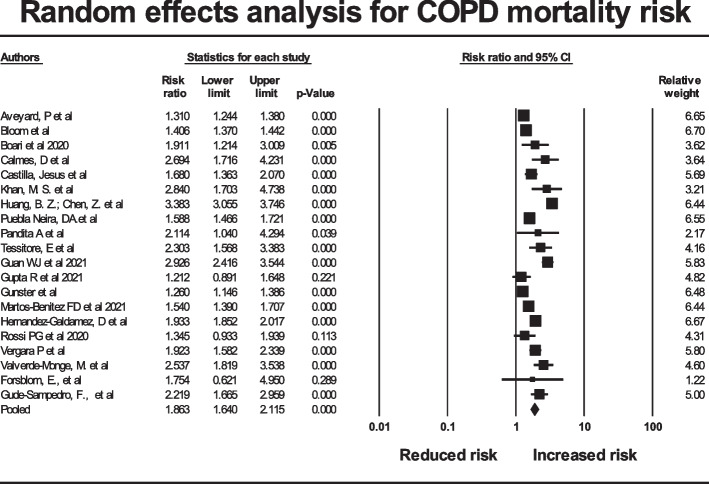
Forest plot for studies in COPD mortality risk analysis. A risk ratio of 1 indicates no increased mortality risk with COPD. Risk ratios greater than 1 represent increased risk. The black diamond gives the pooled risk ratio and the width of the 95% confidence interval

The pooled risk ratio by random effects analysis was 1.863 (95% CI 1.640–2.115) (*p* < 0.001). The prediction interval (the true risk ratio for 95% of comparable studies) lies in the interval 1.09 to 3.20. The forest plot for the studies is given in Fig. 5, with the diamond symbol at the bottom giving the pooled estimate for risk ratio Assessment for publication bias was performed by inspection of the funnel plot and Duval and Tweedie’s trim and fill. On this occasion, the pooled result indicated an increased risk, so missing studies were sought to the left of the mean (so that imputed studies would tend to reduce the observed risk). With 6 studies trimmed, the adjusted risk ratio was 1.585 (95% CI 1.395 to 1.801), so no important effect on the outcome was evident (Supplementary Fig. 4).

Meta-regression using proportion male as the regressor was not significant, with R^2^ analogue of 0%. Using age (mean or median, in years) as a regressor (studies *n* = 17) gave a small but statistically significant log risk ratio coefficient of − 0.0165 (*p* = 0.009): the test of the model gave Q of 6.88, df 1, R^2^ analogue of 25%. Using subregion as a regressor by itself was performed, and the test of the model was not statistically significant (Q = 8.63, df 5, *p* = 0.125). Using both age and subregion as regressors gave an improved model over age alone (studies *n* = 17). Testing the model gave Q of 19.22, df 6, *p* = 0.0038, and R^2^ analogue of 40%. However, the only individual coefficient for subregion that reached statistical significance was Central America (*p* = 0.0136), based on only two studies.

## Discussion

This study had the following aims: for both asthma and COPD as co-morbidities, to determine regional variation in the prevalence of those co-morbidities in patients hospitalized with COVID-19, and to assess the relative risk of death in patients hospitalized with COVID-19 with respect to those co-morbidities, using the same search strategy, inclusion criteria, and analysis for both asthma and COPD.

There are several possible causes of bias in assigning a diagnosis of asthma or COPD when comparing countries: access to healthcare, access to diagnostic facilities, and local guidelines for diagnosis are among them. There is also a risk when comparing background rates of prevalence with prevalence rates in hospitalized patients compared with patients in the community, given there may be different degrees of assiduity in documenting background co-morbidities, perhaps giving rise to spurious associations. In addition, comparisons of mild versus severe cases of COVID-19 infection involves subjective parameters such as severe dyspnoea, making study inclusion open to the same subjectivity. We avoided these sources of bias by looking only at hospitalized patients, whose co-morbidities were recorded on admission to hospital, and are therefore uninfluenced by subsequent severity of illness or death, and looking only at death from any cause as the outcome of interest. Regardless of local rates of recorded prevalence, any increased predisposition to death from COVID-19 would still be evident, since the co-morbidities had been determined prior to knowledge of the outcome.

For asthma, we found large variations in the prevalence in hospitalized patients, from 11.4% in North American studies, to 1.7% in East Asian studies. This is consistent with a previous meta-analysis: variation in asthma prevalence in hospitalized patients varied from 0 to 20% [69]. This is presumably related to background differences in population prevalence of asthma. The findings of a cross-sectional world health survey of doctor-diagnosed asthma in adults gave a regional prevalence of 3.24% in South East Asia, prevalence in China of 0.19%, prevalence in the UK, Sweden and the Netherlands varying between 21.6 to 22.7%, and prevalence in Ecuador of 2.03% [64]. In our analysis, there was a strong correlation between the estimated national prevalence of asthma where the studies had been conducted and the prevalence of asthma in the hospitalized patients.

It must be stated that this variation in asthma prevalence means that the global figure from this meta-analysis of 6.5% has no meaning. The first reason is that some regions of the world were not represented at all in studies included in the analysis. The second reason is that even if we confine our attention to the regions included in the analysis, the number of studies included by region represents published studies from those regions: they are not a random sample, and are not a weighted globally representative sample. This consideration applies equally to other published meta-analyses.

The analysis of the relative risk or odds ratios for asthma for mortality in patients hospitalized with COVID-19 showed significant heterogeneity, but it was not accounted for by region of study origin, and it is therefore reasonable to generalise the outcomes. The pooled risk ratio was 0.918 using a random effects model. If one considers that despite the heterogeneity, we are estimating some common risk applicable globally, then the presence of asthma has not been shown to affect the risk of mortality with COVID-19, with a pooled risk ratio of 0.920. The prediction interval for true effect size in 95% of comparable studies is wide at 0.46 to 1.85 The prediction intervals arise from the random effects analysis allowing for a genuine variation between study results, not accounted for by chance i.e. that there is no one true effect. There are, however, many possibilities which could distort an underlying uniform effect: publication bias, poor methodological design and inadequate analysis, mis-recording or representation of results, and chance. Thus there are limitations to prediction intervals, and the study does not provide any evidence for speculation on causes for heterogeneity (other than by regressing against factors shown to be causing dispersal).

These results support the proposition that in general asthma does not predispose to mortality from COVID-19. This is consistent with most previous studies. An early study in 163 patients with asthma hospitalized with COVID-19 in patients under 65 showed no association with mortality [70]. In the meta-analysis by Sunjaya et al., the risk ratio for mortality for patients with asthma was 0.94 (CI 0.76 to 1.17) [69]. Morais-Almeida et al. performed a literature review, and concluded that there was no strong evidence to support asthma as predisposing to more severe disease in COVID-19, and they also noted the global variation in prevalence [71]. A more recent review by Adir et al. also concluded that asthma per se did not predispose to more severe COVID-19 disease [72].

For COPD, we found marked regional variation in prevalence, with a much lower prevalence in East Asia than in the rest of the world (Table 2). Data for country-specific COPD prevalence is not as readily available as for asthma, and we did not attempt to compare COPD prevalence for hospitalised patients against prevalence in the adult community. In a WHO publication, COPD prevalence by WHO region was noted to vary markedly, and the heterogeneity was largely unexplained, although the authors speculated variation in smoking habit and industrialization might partially explain differences [73]. The overall prevalence in Southeast Asia was 8.8%, in contrast with 13.3% in Europe and 14.5% in the Americas: the authors noted lack of data from key regions. Again, and for the same reasons given for asthma, a global figure for COPD prevalence in hospitalised patients with COVID-19 cannot be derived from this meta-analysis, and it is clear that regional comparisons which involve estimations of COPD prevalence must be undertaken with care and are at risk of being misleading.

For COPD mortality, there was an unequivocal signal that COPD as a co-morbid condition predisposed to increased mortality from COVID-19 in hospitalised patients. The pooled risk ratio was 1.863, CI 1.526 to 1.582. The prediction interval was also consistent with an increased mortality. This is in line with previous data. An editorial early in the course of the pandemic by Leung et al. [74] stated that there is increasing evidence that COPD is a risk factor for more severe COVID-19 disease. In a meta-analysis published in 2021 by Li et al. [75], of retrospective cohort studies looked at the prevalence of COPD in severe cases of COVID-19 compared with non-severe cases. The inclusion criteria for severe cases were wide, including severe dyspnoea, very low oxygen saturation levels, respiratory distress, ICU admission and death. The pooled odds ratio for COPD in severe disease was 2.88 (95% CI 1.89–4.38). Meta-regression using age and region did account for some of the variability in this outcome, but the coefficients for region did not reach statistical significance.

The possible mechanisms by which COPD could increase mortality related to COVID-19 disease have been reviewed elsewhere [76]. In contrast to patients with mild asthma, there are obvious deficiencies in respiratory reserve in patients with COPD who have chronic hyperinflation, emphysema and largely irreversible airways obstruction. There may be some confounding with other co-morbidities related to smoking despite attempts to control for these parameters. There is some evidence for an increased risk for micro-thrombosis. There is evidence of T-cell dysfunction in COPD with reduced cytokine production, and lower alveolar macrophage expression of IFN beta, and this may lead to a sub-optimal host defence response to COVID-19 infection.

There are limitations to this meta-analysis. The first point to make is that for international comparisons of disease prevalence rates, the analysis is dependent on a sufficient number of large studies from a wide range of countries. If the prevalence rates between countries or regions show marked variation, as is the case for both comorbid conditions examined in this analysis, the pooled global prevalence rates become meaningless. Some weight can be attached to pooled regional estimates, provided that the degree of variation is modest. Secondly, the meta-regression does not control for within-study confounding factors such as age and co-morbidities such as cardiovascular disease. It is reassuring that where such interactions were sought in individual studies, the direction of association was not altered e.g. in the study of Moschovis et al. [42] asthma was associated with decreased COVID-19 severity among older adults. Thirdly, the time-frame of publication of the studies included was from 2019 to November 2021. During this period, the original COVID-19 was supplanted by the alpha variant by January 2021, and by the delta variant by mid-2021. The omicron variant arose after the period of this review, so any extrapolation of the results of this meta-analysis to the later variants must be cautious. Although the omicron variant has a lower mortality, a study by Manchanda et al. has compared the effect of co-morbidities on mortality between the delta and omicron variants, and found that COPD increased mortality for both variants to a similar degree [77]. In a study from Italy looking at 65 patients admitted to intensive care with COVID-19 infection, a comparison was made between the prevalence of pulmonary disease (a composite of COPD and pulmonary fibrosis) in patients with delta and omicron variants: the frequency in the omicron group was 35.3% compared with 9.7% in the delta group (*p* = 0.03) [78]. It is unlikely that vaccination will have materially affected the results of this study: the lag-time between acquiring data and publication is usually at least 3 months, most of the data was from 2019 to 2020, and by April 2021 a total globally of 820 million doses of vaccine had been given (likely representing about half that number of people vaccinated with two doses).

## Conclusions

Both for asthma and COPD, prevalence in patients hospitalized with COVID-19 varies markedly by region, and at least for asthma there is a correspondence between the reported national population prevalence and that observed in the hospitalized population. We found no evidence that asthma predisposed to increased mortality in COVID-19 disease. COPD predisposed to clinically and statistically significant increased mortality in patients hospitalised with COVID-19.

### Supplementary Information

**Additional file 1: Supplementary table 1.** Studies included in analysis of asthma prevalence.

**Additional file 2: Supplementary table 2.** Studies included in analysis of COPD prevalence.

**Additional file 3: Supplementary Figure 1.** Funnel plot of asthma prevalence analyzed by random effects, showing raw data and results of Duval and Tweedie’s trim and fill.

**Additional file 4: Supplementary Figure 2.** Funnel plot of study standard error against log risk ratio for asthma mortality.

**Additional file 5: Supplementary figure 3.** Funnel plot of COPD prevalence.

**Additional file 6: Supplementary figure 4.** Funnel plot of study standard error against log risk ratio for COPD mortality.

## Data Availability

The data in this study is extracted from journal articles which have been referenced in the manuscript and are within the public arena.

## Abbreviations

COVID-19: Coronavirus 19
Sars-Cov-2: Severe Acute Respiratory Syndrome Coronavirus-2
HCoVs: Human coronaviruses
