# Supplementary material for: Asthma and COPD as co-morbidities in patients hospitalised with Covid-19 disease: a global systematic review and meta-analysis
Source: BMC Pulm Med. 2023 Nov 22;23:462. doi: 10.1186/s12890-023-02761-5 (PMC10664669; doi:10.1186/s12890-023-02761-5)
Supplement: Supplementary file 1 — Additional file 1: Supplementary table 1. Studies included in analysis of asthma prevalence. [file 12890_2023_2761_MOESM1_ESM.pdf]

Supplementary table 1. Studies included in analysis of asthma prevalence.

Author	Country	Subregion	Study design	Proportion Male	Male (N)	COVID-19 patients total	COVID-19 patients with Asthma	Mean age
Abayomi, A [61] et al	Nigeria	Sub-Saharan	Retrospective cohort	0.66	1379	2075	42	*
Alberca, Ricardo [8] et al	Brazil	S Amer	Retrospective cohort	*	*	215	5	*
Arslan, Yakup [9] et al	Turkey	W Asia	Retrospective cohort	0.49	374	767	58	51.99
Aveyard [62] et al 2021	UK	N Euro	Population cohort	0.56	8037	14479	2266	69.9
Bergman J [11] et al 2021	Sweden	N Euro	Retrospective registry-based	0.59	9433	16083	1419	64
Barroso, B., M. [10] et al	Spain	S Euro	Retrospective Cohort study	0.58	109	189	11	67.52
Beurnier, Antoine [12], et al	France	W Euro	Retrospective Cohort study	*	*	768	37	*
Bloom, Chloe I. [13] et al	UK	N Euro	retrospective cohort study	0.56	41781	74603	7785	71.29
Calmes, Doriane [15] et al	Belgium	W Euro	Retrospective Cohort study	0.49	294	596	57	58.8
Caminati M [16] et al	Italy	S Euro	retrospective	*	*	2000	42	*
Castilla, Jesus [17] et al	Spain	S Euro	Retrospective Cohort study	0.52	1080	2080	147	*
Chudasama [18] et al 2021	UK	N Euro	Observational	0.58	981	1706	226	71
Clark, Chantal C. [19] et al	Netherlands	W Euro	Retrospective Cohort study	0.57	45	79	10	67
Duanmu, Youyou [22] et al	US	N Amer	Cross-sectional study	0.54	13	24	3	64

Forsblom, E. [] et al	Finland	N Euro	Retrospective Cohort study	0.54	316	585	95	57
Gabrielli, Maurizio [24] et al	Italy	S Euro	Retrospective Cohort study	*	*	579	6	*
Guan, Wei-jie [27] et al	China	E Asia	Retrospective Cohort study	0.50	19670	39420	244	55.7
Gude-Sampedro, F. [28] et al	Spain	S Euro	Retrospective Cohort study	0.53	1324	2492	103	70.19
Ho, K. S. [32] et al	US	N Amer	Retrospective Cohort study	0.56	2738	4902	233	64.99
Ko, Jean Y. [37] et al	US	N Amer	surveillance/Retrospective Cohort	0.53	2847	5416	738	55
Lemus Calderon [38] et al	Spain	S Euro	Retrospective Cohort study	*	*	2164	131	*
Martos-Benítez, F. D. [40] et al	Mexico	C Amer	Retrospective Cohort study	*	*	15305	367	44
Newton S [43] et al 2020	US	N Amer	Retrospective series	*	*	443	67	62.66
Nystad, Wenche [44] et al	Norway	N Euro	Retrospective Cohort study	*	*	1025	127	*
Pandita et al [] 2021	US	N Amer	retrospective cohort study	0.53	138	259	30	62
Rosenthal, J. A. [45] et al	US	N Amer	Retrospective Cohort study	*	*	403	60	*
Shin, Eun Kyong [49] et al	Korea	E Asia	Retrospective Cohort study	0.41	2304	5571	128	*
Silver, Victoria [50] et al	US	N Amer	Retrospective Cohort study	0.44	110	249	49	59.6
Terada, M. [52] et al	Japan	E Asia	Retrospective Cohort study	0.61	2063	3376	166	*
Valverde-Monge, M [54] et al	Spain	S Euro	Retrospective Cohort study	0.50	1275	2539	113	62.66

Villamañán, E [] et al	Spain	S Euro	Cross-sectional study	0.57	186	327	13	64.4
Yoshida, Yilin [56] et al	US	N Amer	Prospective cohort study	0.47	367	776	83	60.5
Zhao [60] et al	China	E Asia	Cross-sectional study	0.47	466	1000	12	61
Corradini E [20] et al 2021	Italy	S Euro	Retrospective Cohort study	0.64	1961	3044	116	67
Gimeno-Miguel A [25] et al	Spain	S Euro	Retrospective Cohort study	0.53	3107	5885	335	71.6
Gupta R [30] et al 2021	US	N Amer	Retrospective cohort study	0.54	286	529	30	70
Huang BZ [34] et al 2021	US	N Amer	Retrospective cohort study	*	*	4082	484	*
Ludwig M [39] et al 2021	Germany	W Euro	Retrospective Cohort study	0.54	1265	2343	290	62
Moschovis PP [42] et al 2021	US	N Amer	Retrospective Cohort study	0.57	795	1391	182	59
Puebla Neira DA [46] et al 2021	US	N Amer	Retrospective Cohort study	0.53	16823	31526	3651	72
Hernandez-Galdamez, D [31] et al	Mexico	C Amer	Retrospective cohort study	*	*	23084	1501	*
Soria ME [51] et al 2021	Spain	S Euro	Retrospective Cohort study	0.51	171	338	24	75.26

Note on table: superscripts in author column give the reference for the study specified. Asterisks indicate that data could not be extracted from the study for the parameter tabulated.
