# Supplementary material for: Asthma and COPD as co-morbidities in patients hospitalised with Covid-19 disease: a global systematic review and meta-analysis
Source: BMC Pulm Med. 2023 Nov 22;23:462. doi: 10.1186/s12890-023-02761-5 (PMC10664669; doi:10.1186/s12890-023-02761-5)
Supplement: Supplementary file 2 — Additional file 2: Supplementary table 2. Studies included in analysis of COPD prevalence. [file 12890_2023_2761_MOESM2_ESM.pdf]

Supplementary table 2: Studies included in analysis of COPD prevalence

Author	Country	UN Sub-region	Study design	Proportion Male	Male (N)	COVID-19 patients total	COVID-19 patients with COPD	Mean Age
Bergman J [11] et al 2021	Sweden	N Euro	Retrospective registry-based	*	*	16083	1747	64
Bloom, CI [13] Drake, TM et al	UK	N Euro	Prospective cohort study	*	*	74603	10 266	77.8
Boari, GE [14] Chiarini, G et al	Italy	S Euro	Retrospective cohort	0.67	173	258	35	71
Newton,S [43] et al	US	N Amer	Cohort study	0.49	219	443	46	62.66
Nystad, W [44] Hjellvik, V et al	Norway	N Euro	Cohort study	*	*	1025	67	
Pandita [44] et al	US	N Amer	Retrospective cohort	0.53	138	259	25	62
Aveyard, P [62] et al	UK	N Euro	Population cohort study	0.56	8038	14479	1555	69.9
Calmes, D [15] ; Graff, S et al	Belgium	W Euro	Electronic medical record	0.49	294	596	46	58.8
Castilla, J [17] et al	Spain	S Euro	prospective population-based cohort	0.52	1080	2080	195	
Chudasama, YV [18] et al	UK	N Euro	Observational study	0.58	981	1706	14	71
Corradini, E [20] et al	Italy	S Euro	Electronic medical records	0.64	1961	3044	314	67

Gimeno-Miguel, A [25] et al	Spain	S Euro	Retrospective, observational study	0.53	3107	5885	392	71.6
Guan WJ [27] Liang, W et al	China	E Asia	Retrospective Cohort	0.50	19655	39420	636	55.7
Gupta, R [30] Agrawal, R et al	US	N Amer	Retrospective cohort	0.54	286	529	36	70
Gude-Sampedro, F [28] et al	Spain	S Euro	Retrospective Cohort study	0.53	1324	2492	106	70.19
Hu, X [33]; Hu C, Yang Y et al	China	E Asia	Retrospective cohort	0.48	102	213	4	44
Huang, BZ [34] Chen, Z et al	US	N Amer	Retrospective study	*	*	4082	194	43.6
Khan, MS [35] Dogra, R et al	US	N Amer	Retrospective observational cohort	0.48	224	470	87	*
Gunster C [29] et al 2021	Germany	W Euro	Observational study	0.53	4641	8679	1188	68.6
Ludwig, M [39] Jacob, J et al	Germany	W Euro	Observational study	0.54	1265	2343	306	62
Martos-Benítez, FD [40] et al	Mexico	C Amer	Retrospective analysis	*	*	15305	626	44
Moschovis, PP [42] Lu, M et al	US	N Amer	Retrospective analysis	0.57	795	1391	143	59
Puebla N [46] Daniel A et al	US	N Amer	Retrospective cohort	0.53	16823	31526	4758	72

Hernandez-Galdamez, D [31] et al	Mexico	C Amer	Cross sectional study	*	*	23084	2358	*
Rossi, PG [26] Marino, M et al	Italy	S Euro	Prospective cohort	0.61	657	1075	91	62.19
Shin, EK [49] et al	Korea	E Asia	Retrospective cohort	0.41	2304	5571	40	*
Soria, MG [51] Corton M et al	Spain	S Euro	Retrospective	*	*	338	12	*
Tessitore, E [53] et al	Switzerland	W Euro	Observational	0.54	453	839	49	67
Vergara, P [55] Rossi, L et al	Italy	S Euro	Retrospective observational cohort	0.68	711	1049	102	71
Xiong, T-Y [57] Huang, F-Y et al	China	E Asia	Retrospective study	0.53	250	472	6	43
Zhang, JJ [59] Dong, X et al	China	E Asia	Electronic medical records	0.51	71	140	2	57
Silver V et al [50]	US	N Amer	Retrospective Cohort study	0.44	110	249	32	59.6
Valverde-Monge, M [54] et al	Spain	S Euro	Retrospective Cohort study	0.50	1275	2539	89	62.66
Villamañán, E. [56] et al	Spain	S Euro	Cross-sectional study	0.57	186	327	42	64.4
Yoshida, Yilin [58] et al	US	N Amer	Prospective cohort study	0.47	367	776	140	60.5
Zhao M [60] et al	China	E Asia	Cross-sectional study	0.47	466	1000	23	61
Forsblom, E [23] et al	Finland	N Euro	Retrospective Cohort study	0.54	316	585	15	57

Ko, Jean Y [37] et al	US	N Amer	Surveillance/Retrospective Cohort	0.53	2847	5416	328	55

Note: superscript gives reference number for study specified. Asterisks indicate that data could not be extracted from the study for the parameter tabulated.
