# Supplementary material for: Asthma and COPD as co-morbidities in patients hospitalised with Covid-19 disease: a global systematic review and meta-analysis
Source: BMC Pulm Med. 2023 Nov 22;23:462. doi: 10.1186/s12890-023-02761-5 (PMC10664669; doi:10.1186/s12890-023-02761-5)
Supplement: Supplementary file 3 — Additional file 3: Supplementary Figure 1. Funnel plot of asthma prevalence analyzed by random effects, showing raw data and results of Duval and Tweedie’s trim and fill. [file 12890_2023_2761_MOESM3_ESM.docx]

Supplementary Figure 1: Funnel plot of asthma prevalence analyzed by random effects, showing raw data and results of Duval and Tweedie’s trim and fill.

Under the random effects model the point estimate and 95% confidence interval for the combined studies is 0.0655 (0.0551 to 0.0776) (represented by open diamond on the logit scale). Using Trim and Fill the imputed point estimate is 0.0487 (0.0487 to 0.0738) (represented by black diamond). Three studies (black dots) have been imputed to the left of the mean: the studies trimmed have low standard error, so this is not a small study effect: there is significant underlying heterogeneity. The heterogeneity is reduced by meta-regression (see main text).
