# Supplementary material for: Asthma and COPD as co-morbidities in patients hospitalised with Covid-19 disease: a global systematic review and meta-analysis
Source: BMC Pulm Med. 2023 Nov 22;23:462. doi: 10.1186/s12890-023-02761-5 (PMC10664669; doi:10.1186/s12890-023-02761-5)
Supplement: Supplementary file 4 — Additional file 4: Supplementary Figure 2. Funnel plot of study standard error against log risk ratio for asthma mortality. [file 12890_2023_2761_MOESM4_ESM.docx]

Supplementary Figure 2. Funnel plot of study standard error against log risk ratio for asthma mortality.

Legend: open circles represent studies analyzed, solid circles represent imputed studies. Open diamond represents pooled log risk ratio, closed diamond represents pooled log risk ratio including imputed studies.

With 4 studies imputed to the right of the mean, the point estimate for the pooled point estimate for risk ratio remained less than 1.
