# Supplementary material for: Asthma and COPD as co-morbidities in patients hospitalised with Covid-19 disease: a global systematic review and meta-analysis
Source: BMC Pulm Med. 2023 Nov 22;23:462. doi: 10.1186/s12890-023-02761-5 (PMC10664669; doi:10.1186/s12890-023-02761-5)
Supplement: Supplementary file 5 — Additional file 5: Supplementary figure 3. Funnel plot of COPD prevalence. [file 12890_2023_2761_MOESM5_ESM.docx]

Supplementary figure 3: Funnel plot of COPD prevalence.

Single imputed study in black on left of plot, with negligible effect on estimate of prevalence (observed values point estimate is 0.0659, with adjusted values it is 0.0638). By the same analysis, no missing studies to right of plot. Marked heterogeneity demonstrated.
