# Supplementary material for: Asthma and COPD as co-morbidities in patients hospitalised with Covid-19 disease: a global systematic review and meta-analysis
Source: BMC Pulm Med. 2023 Nov 22;23:462. doi: 10.1186/s12890-023-02761-5 (PMC10664669; doi:10.1186/s12890-023-02761-5)
Supplement: Supplementary file 6 — Additional file 6: Supplementary figure 4. Funnel plot of study standard error against log risk ratio for COPD mortality. [file 12890_2023_2761_MOESM6_ESM.docx]

Supplementary figure 4. Funnel plot of study standard error against log risk ratio for COPD mortality.

Legend: open circles represent studies analyzed, solid circles represent imputed studies. Open diamond represents pooled log risk ratio, closed diamond represents pooled log risk ratio including imputed studies. 6 studies are imputed to the left of the mean, with no major effect on the point estimate. A log risk ratio of 0 would represent no increased risk. The trimmed studies have low standard errors, so there is no evidence of a small study effect.
